# Forward planned intensity modulated radiotherapy (IMRT) for whole breast postoperative radiotherapy. Is it useful? When?

**DOI:** 10.1120/jacmp.v12i2.3451

**Published:** 2011-01-31

**Authors:** Alessio G. Morganti, Savino Cilla, Andrea de Gaetano, Simona Panunzi, Cinzia Digesù, Gabriella Macchia, Mariangela Massaccesi, Francesco Deodato, Gabriella Ferrandina, Numa Cellini, Giovanni Scambia, Angelo Piermattei, Vincenzo Valentini

**Affiliations:** ^1^ Radiotherapy Unit Department of Oncology “John Paul II” Center for High Technology Research and Education in Biomedical Sciences Campobasso; ^2^ Medical Physics Unit Department of Oncology “John Paul II” Center for High Technology Research and Education in Biomedical Sciences Campobasso; ^3^ Gynaecology Oncology Unit Department of Oncology “John Paul II” Center for High Technology Research and Education in Biomedical Sciences Campobasso; ^4^ CNR‐Institute of Systems Analysis and Computer Science (IASI) BioMathLab Rome; ^5^ Department of Radiotherapy Policlinico Universitario “Agostino Gemelli”, Catholic University Rome Italy; ^6^ Gynecology Oncology Department Policlinico Universitario “Agostino Gemelli”, Catholic University Rome Italy

**Keywords:** forward IMRT, breast cancer, whole breast radiotherapy, dosimetry

## Abstract

The purpose was to compare the dosimetric results observed in 201 breast cancer patients submitted to tangential forward intensity‐modulated radiation therapy (IMRT) with those observed in 131 patients treated with a standard wedged 3D technique for postoperative treatment of whole breast, according to breast size and supraclavicular node irradiation. Following dosimetric parameters were used for the comparison: $D_{\text{max}},D_{\text{min}},D_{\text{mean}},V_{95\%}$ and $V_{107\%}$ for the irradiated volume; $D_{\text{max}},D_{\text{mean}},V_{80\%}$ and $V_{95\%}$ for the ipsilateral lung; $D_{\text{max}},D_{\text{mean}},V_{80\%}$ and $V_{95\%}$ for the heart. Stratification was made according to breast size and supraclavicular (SCV) nodal irradiation. As respect to irradiated volume, a significant reduction of $V_{107\%}$ (mean values: 7.0±6.6 versus 2.4±3.7,p<0.001) and $D_{\text{max}}$ (mean % values:111.2±2.7 versus 107.7±6.3,p<0.001), and an increase of $D_{\text{min}}$ (mean % values: 65.0±17.4 versus 74.9±12.9,p<0.001) were observed with forward IMRT. The homogeneity of dose distribution to target volume significantly improved with forward IMRT in all patient groups, irrespective of breast size or supraclavicular nodal irradiation. When patients treated with supraclavicular nodal irradiation were excluded from the analysis, forward IMRT slightly reduced $V_{80\%}$ (mean values: 3.7±2.6 versus 3.0±2.4,p=0.03) and $V_{95\%}$ (mean values 1.9±1.8 versus 1.2%±1.5;p=0.001) of the ipsilateral lung. The dose to the heart tended to be lower with IMRT but this difference was not statistically significant. Tangential forward IMRT in postoperative treatment of whole breast improved dosimetric parameters in terms of homogeneity of dose distribution to the target in a large sample of patients, independent of breast size or supraclavicular nodal irradiation. Lung irradiation was slightly reduced in patients not undergoing to supraclavicular irradiation.

PACS numbers: 87.53.Kn; 87.55.de

## I. INTRODUCTION

Breast cancer is the most common cancer in women. Most are detected at an early stage and often managed with conservative surgery. Postoperative radiotherapy is known to be the standard of care after conservative surgery for early stage breast cancer, as demonstrated by many randomized controlled studies.^(^ ^1^ ^–^ ^3^ ^)^ In these patients, risks of late complications must be strongly considered due to the long expected disease‐free interval. Adjuvant conventional radiotherapy after breast conserving surgery is based on a tangential technique with two photon beams targeting the residual breast. Wedge filters modulating radiation fluence across the beams are commonly used to compensate for dose distribution inhomogeneity due to the irregular shape of mammary gland. Wedge filter modulation is of the same entity along the longitudinal axis of the beam, so that the breast is assumed to be shaped like a hemicylindre. This simplified approximation obviously leads to an inhomogeneous dose distribution within the gland, particularly at the nipple, and in the most superior and inferior portions of fields. The dose within the planning target volume can vary by as much as 27% in some patients^(4)^ and a significant portion of the breast tissue may receive 110% of the prescription dose, with potential hot spots of up to 120%.^(5)^ This heterogeneity may result in increased normal tissue toxicity and poor cosmetic results.^(^ ^6^ ^,^ ^7^ ^)^ In addition, physical compensators were found to increase scatter dose to the contralateral breast.^(8)^ Irradiation of breast is challenging, not only because its irregular shape makes it difficult to achieve a homogeneous dose distribution, but also because of its proximity to organs at risk (OARs), such as the heart and the lung. With both the intent of further optimize dose homogeneity through the breast and improve OARs sparing, multileaf collimator (MLC)‐ based intensity‐modulated radiation therapy (IMRT) (hereafter shown as IMRT) has been tested in several dosimetric studies,^(^ ^9^ ^–^ ^29^ ^)^ with no uniform results. Almost general agreement exists about improved dose homogeneity within the breast with IMRT.^(^ ^9^ ^–^ ^12^ ^,^ ^14^ ^,^ ^16^ ^,^ ^19^ ^,^ ^21^ ^,^ ^22^ ^,^ ^24^ ^,^ ^28^ ^,^ ^29^ ^)^ Target coverage was found to be either improved with IMRT^(^ ^10^ ^,^ ^11^ ^,^ ^13^ ^,^ ^22^ ^,^ ^29^ ^)^ or not significantly modified. ^(^ ^9^ ^,^ ^16^ ^,^ ^17^ ^,^ ^21^ ^)^ Several investigators reported IMRT to improve heart sparing,^(^ ^9^ ^–^ ^16^ ^,^ ^19^ ^,^ ^22^ ^,^ ^23^ ^,^ ^27^ ^)^ while others do not.^(12)^ Dose distribution within the lung was reported to be improved^(^ ^9^ ^–^ ^11^ ^,^ ^16^ ^,^ ^19^ ^,^ ^20^ ^,^ ^21^ ^,^ ^27^ ^,^ ^28^ ^)^ or not significantly modified^(^ ^12^ ^,^ ^13^ ^,^ ^22^ ^,^ ^29^ ^)^ with IMRT. However most of published dosimetric comparisons between IMRT and standard techniques were performed in small groups of 5–43 selected patients.^(^ ^9^ ^–^ ^28^ ^)^ In particular, many studies included only patients with left‐sided breast cancer,^(^ ^10^ ^,^ ^11^ ^,^ ^13^ ^–^ ^16^ ^,^ ^19^ ^,^ ^22^ ^,^ ^23^ ^)^ with large breasts,^(^ ^12^ ^,^ ^18^ ^)^ or with target volume encompassing regional nodes.^(^ ^11^ ^,^ ^13^ ^,^ ^22^ ^)^ In some studies, patients with left‐sided breast carcinoma where selected only if a maximum heart distance of at least 1 cm^(^ ^14^ ^,^ ^16^ ^)^ or 2 cm^(13)^ was measured.

However, to the best of our knowledge, no dosimetric comparisons between IMRT and standard three‐dimensional conformal radiotherapy (3DCRT) with wedge filters were performed in large unselected groups of patients. In particular, no studies aiming to identify patient subgroups where the use of IMRT could be more beneficial have been performed.

Forward IMRT for breast irradiation has been previously described by other investigators.^(24)^ It is a very simplified form of IMRT with only a few segments per field, whose shape and weight are optimized by the dosimetrist in order to achieve the best homogenous dose distribution to the target. Gulybán A et al.^(29)^ conducted a dosimetric comparison between this multisegmented conformal radiation therapy and 3DCRT with weight‐optimized medial and lateral open fields in a large group of unselected patients. They concluded that multisegmented conformal radiation therapy provided a better target coverage than 3DCRT with open fields. Preliminary evaluation of acute toxicity of forward IMRT for breast irradiation together with a simple dosimetric comparison of dose distribution within the target in the overall patient population has been recently published by our group.^(30)^ The aim of the present analysis is a more detailed comparison between 3DCRT with wedge filters and forward IMRT with respect to the impact on target and OARs irradiation. The size of breast has been previously reported to impact on homogeneity of dose distribution, with standard 3DCRT.^(31)^ An evaluation of influence of breast size on dosimetric parameters was carried out. Prophylactic supraclavicular nodal irradiation was also explored as a possible factor conditioning dosimetric results of IMRT versus 3DCRT.

## II. MATERIALS AND METHODS

### A. Treatment planning

In all patients computed tomography (CT) was used for treatment planning. At the time of CT scanning, patients were placed in the supine position, with the ipsilateral arm placed above their heads. An in‐house designed angled board was used as an immobilization device. A radio‐opaque wire was placed in order to delimitate the palpable breast and to assist in target delineation. Contiguous 5 mm CT axial images were obtained extending from the larynx to the upper abdomen, including the entire breasts and lungs bilateral. The CT scans were transferred to a treatment planning workstation (Plato Sunrise, Nucletron B.V., Veenendaal, Netherlands) for definition of target volumes and critical structures and for treatment planning. The irradiated volume included the whole breast excluding the most external cutaneous‐subcutaneous 5 mm (except for pT4 for cutaneous infiltration). Supraclavicular (SCV) region, as outlined by Madu et al.,^(32)^ was also included whenever clinically indicated. The heart and ipsilateral lung were considered as organs at risk. The heart was defined as all the visible myocardium and pericardium, from the apex to the right auricle, atrium and infundibulum of the ventricle, excluding the pulmonary trunk, root of the ascending aorta, superior vena cava and pericardium. The ipsilateral lung contours were generated using an automated threshold‐contouring tool.

The treatment was performed with the tangential technique and slight beam angulation (gantry angles optimized to match divergence of the posterior edges of the beam) to reduce the dose to the OARs. Beam angulation was adapted to avoid contralateral breast irradiation. In patients undergoing also SCV irradiation, a mono‐isocentric technique was used. Caudal to the isocenter, the breast volume was irradiated with the tangential technique. Cranial to the isocenter, the supraclavicular volume was irradiated with two opposed beams. The anterior field was angled 10°–15° to avoid the spinal cord, and the posterior field was matched. The fields were more heavily weighted to the anterior.

In 3DCRT patients, breast irradiation was performed with conformed tangential beams with standard MLC, of suitable energy (usually 6 MV) with wedge filters.

In IMRT patients, a forward “field in field” IMRT technique was used, as we previously described.^(30)^ Briefly, the contribution of each two tangential beams was divided into two different segments. One segment was designed to include the whole breast without filters (usually with 6 MV photons). This configuration, in the absence of filters, results in a volume of underdosage in the thickest region of the breast. A second segment (usually with photons of 15 MV energy in order to increase the dose to the deepest part of the breast while sparing the most superficial part) was directed to this area of underdosage to compensate for dose loss, as follows. From the optimized dose distribution of the three‐dimensional plan with open tangent fields, a dose cloud was derived (individually for each patient) at a dose level between 106% and 109%. The MLC of the second segment was conformed to cover this dose cloud. Approximately 8% to 10% of the prescription dose was delivered with these reduced fields. Plans were normalized and prescribed to a reference point (100% dose) within the target volume. Wedge filter angles for 3DCRT beams and weight of the two adjunctive segments for IMRT beams and reference dose point position were optimized in order to accomplish the best achievable homogeneous target coverage (−5% to +7% of prescribed dose), according to ICRU Report 62 criteria.^(33)^ However irregular shape of breast tissue did not allow for compliance with the minimum (95%) and maximum (107%) dose limit to the target volume in all patients.

For all plans, dose calculation was performed by the pencil beam approach, which is based on pencil kernels.^(34)^ Inhomogeneity correction was applied by the equivalent path length (EPL) method. This means that all depth dependent parameters are evaluated at a depth in water defined by the radiological depth of the calculation point (i.e., the geometrical depth in water where the same attenuation would be obtained). In both techniques, the dose calculation was performed with a dose grid resolution of 0.3 cm.

### B. Dosimetric comparisons

Dose‐volume histograms (DVHs) were generated for all relevant structures for both techniques. The impact of radiotherapy technique was evaluated on a series of parameters. As concern the irradiated volume, maximal $\left(D_{\text{max}}\right)$, minimal $\left(D_{\text{min}}\right)$, and mean dose $\left(D_{\text{mean}}\right)$ and volume of structure receiving at least 95% $\left(V_{95\%}\right)$ and 107% $\left(V_{107\%}\right)$ of prescribed dose, were considered. $D_{\text{max}},D_{\text{mean}},V_{80\%}$ and $V_{95\%}$ were evaluated for ipsilateral lung. $D_{\text{max}},D_{\text{mean}},V_{80\%}$ and $V_{95\%}$ of the heart were also recorded.

On the same parameters, the impact of breast volume (cc) and of regional nodes irradiation was also evaluated.

### C. Statistical analysis

In order to see if the accelerated IMRT‐based postoperative radiotherapy is more efficient than the “standard” 3D postoperative radiotherapy, a comparison was made between the two techniques in terms of dosimetric parameters. Data were analyzed with R software version 2.6.1 (The R Foundation for Statistical Computing, 2007). A t‐test for independent samples was used for comparison. Results are reported in terms of mean values ± standard deviation. The analysis was conducted on the patient global population, and again but this time excluding the patients undergoing prophylactic supraclavicular irradiation. This exclusion allowed a more reliable evaluation of the impact of IMRT in terms of lung irradiation. In fact, this is obviously higher in the patients irradiated on the supraclavicular region.

## III. RESULTS

### A. Patient characteristics

Three hundred and thirty‐two patients underwent whole breast postoperative radiotherapy and were included in the analysis. Table 1 shows characteristics of the sample studied. Patients were divided into two groups: patients who were treated with IMRT‐based postoperative radiotherapy (IMRT‐group: 201 patients), and patients who underwent the “standard” 3D postoperative radiotherapy (3D‐group: 131 patients). Fifty‐two patients received prophylactic irradiation of supraclavicular lymph nodal region (IMRT group: 30/201 patients, 3D‐group: 22/131 patients).

**Table 1:** Patient characteristics.

		*3D‐CRT*		*IMRT*		*All Patients*	
		*N°*	*%*	*N°*	*%*	*N°*	*%*
Total:	131	39.5	201	60.5	332	100
Average Age±SD(yrs):		55.4±12	/	58.5±11	/	57.5±12	/
Cancer Site:	Right Breast	62	47.3	51	51.5	50	49.0
	Left Breast	69	52.7	48	48.5	52	51.0
TNM Stage:	I	66	50.4	103	51.2	169	50.9
	II	52	39.7	79	39.3	131	39.5
	III	13	9.9	19	9.4	32	9.6
Regional Node Irradiation:	Yes	22	16.8	30	14.9	52	15.6
	No	109	83.2	171	85.1	280	84.4

### B. Dosimetric comparison

#### B.1 Target coverage

Mean breast volume was 518 cc (range 43–1818 cc) in overall patient population, 503 cc (range 50–1818 cc) and 528 cc (range 43–1556 cc) in 3DCRT and IMRT group, respectively (p=not significant). Mean thickness of the breast along the posterior field edge was 15.4 cm (range 5.7–26.5 cm). The analyses of the dosimetric variables in overall population showed that there was a significant difference between 3DCRT and IMRT with respect to $D_{\text{max}},D_{\text{min}}$, and $V_{107\%}$ values of the irradiated volume, with IMRT performing better, as described in Table 2. In those patients undergoing regional node irradiation, IMRT reduced $D_{\text{max}}$, as showed in Table 3.

**Table 2:** Target coverage: dosimetric comparison between standard technique and forward‐IMRT (% value) in overall population.

		*3D*			*IMRT*		
*Parameter*	*Mean*	±SD	*Range*	*Mean*	±SD	*Range*	*p*
$D_{\text{max}}$	111.2	2.7	104–117	107.7	1.5	104–111	<0.001
$D_{\text{min}}$	65.0	17.4	4–96	74.9	12.9	38–94	<0.001
$D_{\text{mean}}$	100.7	1.7	91–102	100.6	1.1	98–103	0.512
$V_{95\%}$	97.4	3.8	70–100	96.9	6.2	80–100	0.420
$V_{107\%}$	7.0	6.6	0–44	2.4	3.7	0–20	<0.001

**Table 3:** Target coverage: dosimetric comparison between standard technique and forward‐IMRT (% value), with or without supraclavicular nodal irradiation.

		*With Nodal RT*			*Without Nodal RT*	
	*3D (22 pts)*	*IMRT (30 pts)*		*3D (109 pts)*	*IMRT (171 pts)*	
*Parameter*	Mean±SD	Mean±SD	*p*	Mean±SD	Mean±SD	*p*
$D_{\text{max}}$	109.8±3.0	107.9±1.4	0.011	111.5±2.5	107.2±1.5	<0.001
$D_{\text{min}}$	67.0±14.6	72.6±12.5	0.159	64.6±18.0	75.3±15.7	<0.001
$D_{\text{mean}}$	100.0±1.4	100.6±0.9	0.08	100.8±1.7	100.5±0.9	0.07
$V_{95\%}$	96.9±3.0	95.8±2.3	0.136	97.5±3.9	97.1±6.5	0.551
$V_{107\%}$	3.7±.5	3.1±4.0	0.617	7.7±6.7	2.3±3.6	<0.001

Patients were divided into three group according to breast size (small size: <360 cm^3^; medium: ≥360.0 cm^3^ and ≤568.0 cm^3^; large $>568\;{\text{cm}}^{3}$). As showed in Table 4, IMRT provided better target homogeneity than 3DCRT with wedge filters, in all patient groups.

**Table 4:** Target coverage: dosimetric comparison between standard technique and forward‐IMRT (% value), according to breast size.

		*Small Size Breast* $\left(<360.0\;{\text{cm}}^{3}\right)$		*Medium Size Breast* $(\geq 360.0\;{\text{cm}}^{3}$ *and* $\leq 568.0\;{\text{cm}}^{3}$)			*Large Size Breast* $\left(>568.0\;{\text{cm}}^{3}\right)$		
*Parameter*	3D Mean±SD	IMRT Mean±SD	*p*	3D Mean±SD	IMRT Mean±SD	*p*	3D Mean±SD	IMRT Mean±SD	*p*
$D_{\text{max}}$	110.6±2.6	106.8±1.3	<0.001	111.0±2.3	107.1±1.6	<0.001	111.9±2.9	107.9±1.4	<0.001
$D_{\text{min}}$	67.9±14.9	74.1±12.7	0.02	69.9±15.0	74.8±12.5	0.09	57.9±19.6	76.9±19.3	<0.001
$D_{\text{mean}}$	101.0±1.5	100.7±0.9	0.24	100.6±1.1	100.3±1.1	0.29	100.4±2.2	100.6±0.9	0.59
$V_{95\%}$	97.6±2.2	97.0±5.9	0.48	98.1±1.8	96.7±7.1	0.12	96.5±5.6	96.9±5.2	0.68
$V_{107\%}$	8.3±8.3	1.8±3.3	<0.001	6.1±4.6	2.3±3.6	<0.001	6.3±5.7	3.0±4.1	0.001

### C. Normal tissue sparing

#### C.1 Lung

In overall population, no statistically significant difference in lung sparing was recorded between 3DCRT and IMRT (Table 5). A slight advantage of IMRT (amelioration of $V_{80\%}$ and $V_{95\%}$) was observed when patients treated with supraclavicular nodal irradiation were excluded from the analysis (Table 6).

**Table 5:** Normal tissue sparing: dosimetric comparison between standard technique and forward‐IMRT (% value) for lung and heart in overall patient population.

	*3D*		*IMRT*		
*Parameter*	*Mean*	±SD	*Mean*	±SD	*P*
$D_{\text{max}}$ Lung	99.9	11.6	97.6	10.8	0.059
$D_{\text{mean}}$ Lung	9.8	6	9.1	6.2	0.273
$V_{80\%}$ Lung	4.6	3.8	4.3	4.5	0.621
$V_{95\%}$ Lung	2.5	2.4	2.4	7.2	0.855
$D_{\text{max}}$ Heart^a^	63.0	33.0	57.6	34	0.359
$D_{\text{mean}}$ Heart^a^	3.6	3.3	3.4	2.3	0.648
$V_{80\%}$ Heart^a^	0.8	3.1	0.3	0.8	0.100
$V_{95\%}$ Heart^a^	0.2	0.8	0.1	0.3	0.163

a Analysis on 169 left breast cancer patients

**Table 6:** Normal tissue sparing: dosimetric comparison between standard technique and forward‐IMRT (% value) for lung and heart, with or without prophylactic nodal irradiation.

*Parameter*	*3D (109 pts)* Mean±SD	*Without Nodal RT IMRT (171 pts)* Mean±SD	*p*	*3D (22 pts)* Mean±SD	*With Nodal RT IMRT (30 pts)* Mean±SD	*p*
$D_{\text{max}}$ Lung	99.1±12.5	96.7±11.3	0.104	103.7±2.5	102.3±2.9	0.069
$D_{\text{mean}}$ Lung	8.0±3.8	7.2±3.3	0.066	18.7±6.7	19.7±7.9	0.626
$V_{80\%}$ Lung	3.7±2.5	3.0±2.4	0.034	9.1±5.5	12.0±5.9	0.080
$V_{95\%}$ Lung	1.9±1.8	1.2±1.5	0.001	5.2±3.0	9.2±16.8	0.227
$D_{\text{max}}$ Heart^a^	59.6±32.8	56.4±33.8	0.612	78.2±31.5	66.1±35.2	0.418
$D_{\text{mean}}$ Heart^a^	3.0±2.1	3.2±2.1	0.699	6.3±5.7	5.0±3.2	0.547
$V_{80\%}$ Heart^a^	0.3±0.8	0.2±0.6	0.622	3.3±6.7	0.8±1.4	0.310
$V_{95\%}$ Heart^a^	0.1±0.4	0.1±0.3	0.547	0.7±1.5	0.04±0.09	0.212

a Analysis on 169 left breast cancer patients

As shown in Table 7, in small‐sized breast, IMRT reduced $D_{\text{max}}$ (mean % values: 101.6±4.9 versus 96.8±13.0;p=0.01) and $V_{95\%}$ (mean values: 1.7±1.6 versus 0.9%±1.1%,p=0.009) to the lung. In large‐sized breast, IMRT reduced $D_{\text{mean}}$ (mean % values: 9.7±7.8 versus 7.8±3.6,p=0.02) and $V_{95\%}\;(2.3\pm 2.2$ versus 0.9±1.1,p=0.001) to the lung.

**Table 7:** Normal tissue sparing: dosimetric comparison between standard technique and forward‐IMRT (% value) for lung (cases with supraclavicular irradiation were not included in the analysis) and heart, according to breast size.

	*Small Size Breast* $\left(<360.0\;{\text{cm}}^{3}\right)$			*Medium Size Breast* ($\geq 360.0\;{\text{cm}}^{3}$ *and* $\leq 568.0\;{\text{cm}}^{3}$)			*Large Size Breast* $\left(>568.0\;{\text{cm}}^{3}\right)$		
*Parameter*	3D Mean±SD	IMRT Mean±SD	*p*	3D Mean±SD	IMRT Mean±SD	*p*	3D Mean±SD	IMRT Mean±SD	*p*
$D_{\text{max}}$ Lung	101.6±4.9	96.8±13.0	0.01	95.8±19.6	96.1±14.0	0.94	98.8±11.7	97.1±5.1	0.42
$D_{\text{mean}}$ Lung	7.0±2.9	6.0±2.3	0.09	7.3±3.8	7.6±3.6	0.72	9.7±7.8	7.8±3.6	0.02
$V_{80\%}$ Lung	3.2±2.0	2.5±2.5	0.07	3.5±2.7	3.1±2.2	0.55	4.3±2.8	3.3±3.05	0.13
$V_{95\%}$ Lung	1.7±1.6	0.9±1.1	0.009	1.7±1.4	1.6±1.9	0.96	2.3±2.2	0.9±1.2	0.001
$D_{\text{max}}$ Heart^a^	56.7±33.3	46.8±34.6	0.29	63.5±35.4	±35.1	66.5	0.79±30.7	71.6±30.0	60.2 0.23
$D_{\text{mean}}$ Heart^a^	2.5±2.5	2.2±1.4	0.67	4.8±5.0	4.1±2.7	0.67	4.1±1.8	3.7±2.3	0.59
$V_{80\%}$ Heart^a^	0.9±4.0	0.1±0.5	0.41	1.2±2.8	0.4±0.8	0.31	0.3±0.8	0.3±0.9	0.78
$V_{95\%}$ Heart^a^	0.1±0.5	0.04±0.1	0.49	0.5±1.2	0.1±0.5	0.30	0.1±0.5	0.01±0.08	0.39

a Analysis on 169 left breast cancer patients

#### C.2 Heart

In overall population, no statistically significant difference in heart sparing was recorded between 3DCRT and IMRT (Table 5). Neither prophylactic nodal irradiation (Table 6) nor breast size (Table 7) was influenced.

## IV. DISCUSSION

A dosimetric analysis including 332 patients was performed with the aim to quantify the potential advantage of forward tangent IMRT over standard wedged 3D radiotherapy in postoperative treatment of breast carcinoma. Forward IMRT allowed a more homogeneous dose distribution within the breast than 3DCRT, resulting in smaller volume of breast receiving higher than 107% of prescription dose, lower maximal and higher minimal dose, independently of breast size. Standard deviation was also smaller for IMRT, suggesting potential advantage even in patients with large inhomogeneity with 3DCRT.

Target coverage, as represented by $V_{95\%}$, was found to be similar with both IMRT and 3DCRT techniques. In patients undergoing SCV nodal irradiation, IMRT seems not to significantly improve target coverage or homogeneity except for a reduction in maximal dose to the irradiated breast. A little benefit of IMRT over 3DCRT in lung sparing was observed only in patients who didn't undergo to SCV nodal irradiation and, particularly, in patients with large‐size breast. The two techniques showed no differences with respect to heart irradiation.

Breast IMRT may involve the use of two different modalities for the segment weight definition, namely forward or inverse planning. Mihai et al.^(35)^ compared the two algorithms as respect to the homogeneity of dose distribution and found them to be equivalent. Independently from the type of optimization algorithm used, almost all published studies agree in considering IMRT a method for improving dose homogeneity,^(^ ^9^ ^–^ ^12^ ^,^ ^14^ ^–^ ^16^ ^,^ ^19^ ^,^ ^21^ ^,^ ^22^ ^)^ and our results are in agreement with published ones. This finding could be explained by the fact that MLC allows for a modulation of intensity fluence that is not the same entity amount along the axis of the beam, like it is with wedges. This probably makes it possible to better accomplish for the irregular shape of mammary gland and for interindividual variability. Many clinical experiences supported these dosimetric results. IMRT reduced both acute and late skin toxicity in many clinical experiences of whole breast postoperative radiotherapy,^(^ ^36^ ^–^ ^40^ ^)^ particularly in patients with large breasts.^(41)^ Since an improvement in cosmetic outcome is undoubtedly of interest, the strategy of shortening the overall treatment time with comparable clinical outcome could be attractive as well, particularly in elderly patients or in busy departments. Preliminary data in patients who have been submitted to accelerated forward IMRT whole breast radiotherapy has been recently reported,^(^ ^30^ ^,^ ^42^ ^)^ with results in terms of both cosmetic outcome and tumor control at least comparable to that observed with standard fractionation.

In patients undergoing supraclavicular irradiation, forward IMRT seems not to significantly improve either homogeneity or target coverage. Only maximal dose delivered to the target was appreciably reduced. Because of the use of the same technique for SCV region irradiation in both 3DCRT and IMRT patients, and given that IMRT was found to provide improved dose homogeneity within the breast, this result is probably not completely true. The number of patients in this sample could be too small to allow for the detection of any statistically significant difference between the two groups for $D_{\text{min}}$ and $V_{107\%}$. Nevertheless, the use of an IMRT technique for the irradiation of SCV nodes could be considered as well, in order to further improve dose homogeneity and target coverage, as reported by Dogan et al.^(43)^.

The benefit of IMRT in terms of critical normal structures sparing has been best described for concave structures, such as the chest wall, which wraps around the lung and anterior portion of the heart.^(44)^ However, it's well known that such potential advantage cannot be exploited when two opposite beams, like those tangent ones, are used. Obviously, the use of multiple beam arrangements to irradiate the whole breast, produces a reduction of high dose to normal structure, namely heart^(^ ^10^ ^,^ ^11^ ^,^ ^16^ ^,^ ^22^ ^,^ ^45^ ^)^ and lung.^(^ ^10^ ^,^ ^11^ ^,^ ^16^ ^,^ ^20^ ^,^ ^45^ ^)^ On the contrary, many authors reported that healthy tissue volumes irradiated with low doses, particularly contralateral breast and lung, are increased with IMRT.^(^ ^11^ ^,^ ^45^ ^,^ ^46^ ^)^ However, a tangent arrangement of beams can allow for a reduction of healthy tissue dose even if multiple beams are used.^(10)^ In our study, a forward IMRT with two tangential opposite beams was used. Surprisingly, even high‐dose volume within the ipsilateral lung was minimized with forward tangent IMRT, as compared to conventional wedge 3DCRT. Similar findings were also reported in small series, using a tangent IMRT technique, by other investigators.^(^ ^9^ ^,^ ^27^ ^,^ ^45^ ^,^ ^47^ ^)^ It has already been said that wedge filters are used to compensate for the irregular shape of mammary gland so that their thinnest part is oriented toward the posterior side of the field. Due to the concave shape of chest wall, some volume of lung is expected to enter into the posterior part of the field, exactly where intensity fluence attenuation is of minor entity. As a consequence, an area of relatively high dose within the pulmonary tissue may result. MLC‐based IMRT could allow for a more flexible modulation of intensity fluence, thus resulting in a reduction of high‐dose region within the lung. This effect could be of more relevance in patients with large sized breasts, because in these cases a larger amount of lung tissue is included in the irradiation fields. In our experience, the mean dose delivered to the lung was appreciably reduced with IMRT only in large sized breasts.

A similar reason could explain those findings of improved heart sparing reported by some authors using a tangent IMRT technique.^(^ ^9^ ^,^ ^13^ ^,^ ^15^ ^)^ In our experience, dose to the heart tended to be lower with IMRT but this difference did not reach any statistical significance. No benefit of IMRT in heart sparing was observed either for small nor for large size breast, neither when supraclavicular irradiation was performed nor when it was not. This finding could be at least in part attributable to the unselected nature of our patient population (patients with unfavorable cardiac anatomy were selectively considered in other reports.^(^ ^13^ ^,^ ^15^ ^)^). A difference in the method used for cardiac dose calculation should also be considered.^(9)^

Until dosimetric results of an improved dose distribution within the target with IMRT begin to be corroborated by many clinical findings, the clinical relevance of dosimetric advantage of IMRT in lung and heart sparing remains to be confirmed.

## V. CONCLUSIONS

The use of postoperative forward planned IMRT for whole breast irradiation improved dosimetric parameters in terms of homogeneity to the target, in a large sample of patients independent of breast size. In our opinion, forward tangent IMRT should be preferred over conventional wedge 3DCRT whenever a homogenous dose distribution within the breast is desired. Particularly in patients with large breasts, forward tangent IMRT also provides a slight increased benefit in sparing the ipsilateral lung.
